# Skeletal muscle regeneration failure in ischemic-damaged limbs is associated with pro-inflammatory macrophages and premature differentiation of satellite cells

**DOI:** 10.1186/s13073-023-01250-y

**Published:** 2023-11-10

**Authors:** Kevin W. Southerland, Yueyuan Xu, Derek T. Peters, Xin Lin, Xiaolin Wei, Yu Xiang, Kaileen Fei, Lindsey A. Olivere, Jeremy M. Morowitz, James Otto, Qunsheng Dai, Christopher D. Kontos, Yarui Diao

**Affiliations:** 1 Division of Vascular and Endovascular Surgery, Department of Surgery, Duke University Medical Center, Durham, NC 27710 USA; 2 Department of Cell Biology, Duke University Medical Center, Durham, NC 27710 USA; 3 Duke Regeneration Center, Duke University Medical Center, Durham, NC 27710 USA; 4 Center for Advanced Genomic Technologies, Duke University, Durham, NC 27708 USA; 5 Duke University School of Medicine, Duke University, Durham, NC 27710 USA; 6 Division of Vascular Surgery, Department of Surgery, University of Pittsburgh Medical Center, Pittsburgh, PA 15217 USA; 7 Development and Stem Cell Biology Program, Duke University, Durham, NC 27710 USA; 8 Division of Cardiology, Department of Medicine, Duke University Medical Center, Durham, NC 27710 USA; 9 Department of Orthopaedic Surgery, Duke University Medical Center, Durham, NC 27710 USA; 10 Department of Pathology, Duke University Medical Center, Durham, NC 27710 USA

**Keywords:** Peripheral arterial disease, Chronic limb-threatening ischemia, Single-cell transcriptome analysis, Murine hindlimb ischemia, Skeletal muscle regeneration, Muscle satellite cells, Macrophage polarization

## Abstract

**Background:**

Chronic limb-threatening ischemia (CLTI), a severe manifestation of peripheral arterial disease (PAD), is associated with a 1-year limb amputation rate of approximately 15–20% and substantial mortality. A key feature of CLTI is the compromised regenerative ability of skeletal muscle; however, the mechanisms responsible for this impairment are not yet fully understood. In this study, we aim to delineate pathological changes at both the cellular and transcriptomic levels, as well as in cell–cell signaling pathways, associated with compromised muscle regeneration in limb ischemia in both human tissue samples and murine models of CLTI.

**Methods:**

We performed single-cell transcriptome analysis of ischemic and non-ischemic muscle from the same CLTI patients and from a murine model of CLTI. In both datasets, we analyzed gene expression changes in macrophage and muscle satellite cell (MuSC) populations as well as differential cell–cell signaling interactions and differentiation trajectories.

**Results:**

Single-cell transcriptomic profiling and immunofluorescence analysis of CLTI patient skeletal muscle demonstrated that ischemic-damaged tissue displays a pro-inflammatory macrophage signature. Comparable results were observed in a murine CLTI model. Moreover, integrated analyses of both human and murine datasets revealed premature differentiation of MuSCs to be a key feature of failed muscle regeneration in the ischemic limb. Furthermore, in silico inferences of intercellular communication and in vitro assays highlight the importance of macrophage-MuSC signaling in ischemia induced muscle injuries.

**Conclusions:**

Collectively, our research provides the first single-cell transcriptome atlases of skeletal muscle from CLTI patients and a murine CLTI model, emphasizing the crucial role of macrophages and inflammation in regulating muscle regeneration in CLTI through interactions with MuSCs.

**Supplementary Information:**

The online version contains supplementary material available at 10.1186/s13073-023-01250-y.

## Background

Atherosclerotic vascular diseases that cause tissue ischemia are the cause of pathological conditions such as myocardial infarction, stroke, and peripheral artery disease (PAD) [1–5]. In PAD, the most severe clinical manifestation is chronic limb threatening ischemia (CLTI), which is associated with a high incidence of permanent limb tissue loss [6]. Accordingly, about 15–20% of CLTI patients undergo limb amputation within 1 year of diagnosis, and 50% die within 5 years [7]. The current treatment options for CLTI patients focus primarily on improving limb perfusion but these strategies often fail to prevent disease progression or limb loss, pointing to a mechanism other than simply tissue perfusion as the sole etiology of tissue injury [8]. Accumulating evidence now points to the ability of the skeletal muscle to withstand ischemic injury or to regenerate in the setting of ischemia as important mediators of tissue loss in CLTI [9–13]. In support of this notion is the discrepancy between CLTI patients and those with intermittent claudication (IC), a mild form of PAD. IC presents as reproducible muscle pain with exertion that is relieved with rest [14]. Patients with IC have more favorable clinical outcomes than CLTI patients. In fact, the 1-year limb loss rate for IC is <1% [15]. Intriguingly, a subset of IC patients are found to have atherosclerosis comparable to that of CLTI patients but do not develop the permanent tissue loss phenotype characteristic of CLTI (Additional file 1: Fig S1A, top) [14, 16]. The distinct clinical outcomes of patients with IC versus CLTI suggest that differences in the reparative capacity of skeletal muscle may play a key role in determining the course of disease progression in the two groups. Indeed, skeletal muscle in CLTI limbs often exhibits extensive fibrosis and fatty deposition as well as a distinct mitochondriopathy that distinguishes CLTI from IC skeletal muscle tissue, suggesting a role for pathologic alterations in skeletal muscle regeneration in the development of CLTI [17, 18]. Therefore, understanding the unique cellular and molecular mechanisms involved in ischemia-induced muscle regeneration will likely shed light on the development of new regenerative medicine strategies for limb salvage in CLTI patients that are independent of limb perfusion.

In a pre-clinical murine model of CLTI, in which ligation of the femoral artery causes hind limb ischemia (HLI), the degree of tissue loss is highly strain dependent [19]. Specifically, following HLI surgery, BALB/c mice develop an extensive and irreversible limb tissue loss phenotype while C57BL/6 mice are resistant to tissue loss and initiate a potent muscle regeneration program [13]. Thus, murine models of HLI provide a unique opportunity to study the mechanistic determinants of skeletal muscle regeneration in the context of ischemia. In mammals, successful skeletal muscle regeneration requires the orchestrated activation, proliferation, and differentiation of muscle satellite cells (MuSCs, also known as muscle stem cells) that are normally quiescent in the uninjured state [20, 21]. The regenerative capacity of MuSCs is supported by various cell types that comprise the MuSC niche, including macrophages [22–25]. During skeletal muscle regeneration in response to injury, pro-inflammatory macrophages, mostly derived from the peripheral monocyte [26–28], are enriched at the site of tissue damage. This is followed by a polarization process in which the macrophages acquire an anti-inflammatory and pro-reparative state during the later stages of tissue repair [29, 30]. Inflammatory macrophages, and later, macrophages polarized into a regenerative phenotype, play critical roles in numerous aspects of muscle regeneration, including phagocytosis of tissue debris, assembly of extracellular matrix (ECM), stimulation of angiogenesis, and support for the proliferation and differentiation of MuSCs through the secretion of pro-regenerative factors [22]. Despite the well-established role of macrophages in skeletal muscle regeneration, whether and how macrophages regulate limb tissue repair in response to an ischemic insult in the context of CLTI remains to be elucidated.

Here, through single-cell transcriptome analysis of skeletal muscle tissue from representative CLTI patients and murine models of HLI, we show that non-regenerative, ischemic-injured limbs are enriched with macrophages exhibiting a persistent pro-inflammatory signature. Significantly, these pro-inflammatory macrophages do not express the pro-regenerative cytokines that normally promote the proper balance of proliferation versus myogenic differentiation in MuSCs. Our findings support the idea that macrophages play a critical role in regulating limb regeneration in ischemia-induced tissue damage and provide the first single-cell transcriptome atlas of CLTI, both in humans and mice, as a valuable resource for future studies of CLTI pathobiology and potential regenerative therapies.

## Methods

PCR primers and oligo nucleotides used in this study are listed in Additional file 2: Table S1.

### Human tissue collection and single-cell isolation

Skeletal muscle was obtained from CLTI patients (*n* = 3) undergoing lower-limb amputation in accordance with a research protocol approved by the Duke University Institutional Review Board (IRB#Pro00065709). Paired samples from proximal and distal muscle bodies were collected and subject to mechanical dissociation. For above knee amputations (AKA), the proximal muscle specimen was obtained from the vastus medialis and the distal specimen from the tibialis anterior. For below knee amputations (BKA), proximal and distal specimens were both obtained from the tibialis anterior. A subsequent enzymatic digestion was performed using either 0.05% pronase (Sigma, 537088) for 1 h (Patient #1) or 3.7mg/mL collagenase II (Worthington, LS004177) for 90 min followed by 6 mg/mL dispase (Gibco, 17105-041) for 30 min (Patients #2 and #3). Cells were passed through a 100-uM Steriflip vacuum filter (EMD Millipore, SCNY00100) and resuspended in Ham’s F-10 media supplemented with 10% horse serum and 1× penicillin/streptomycin. The single-cell suspensions were stained with propidium iodide (PI). Fluorescence-activated cell sorting (FACS) was performed using a SonySorter SH800S to isolate PI- live cells. For human samples, approximately 150,000 PI- live cells were sorted per sample and 16,000–24,000 cells used for scRNA-seq library generation.

### Animal protocol, mouse procedures, and euthanasia method

The animal experiments adhered to the protocols established by the Institutional Animal Care and Use Committee (IACUC) at A043-22-03, complying with the National Institutes of Health (NIH) guide for the care and use of laboratory animals. For mouse euthanasia, the mice were placed in the cage within the euthanasia chamber and filled with CO_2_ for at least 4 or 5 min with CO_2_ tank regulator set to displace 30–70% of the cage volume per minute.

### Mouse hind limb ischemia injury and single-cell isolation

Hind limb ischemia (HLI) surgery was performed on male 3-month-old C57BL/6 and BALBc mice. To induce muscle ischemia, the femoral artery was ligated proximally, inferior to the inguinal ligament just proximal to the lateral circumflex femoral artery, as well as distally, immediately proximal to the bifurcation of the popliteal and saphenous arteries [31]. Laser Doppler perfusion imaging (LDPI) was performed with a Moor Instruments LDI2-High Resolution (830nM) System (Moor, Axminster, UK) to quantify and assess blood flow restoration.

### Mouse tissue collection and single-cell isolation

Mouse hindlimb muscles, including TA, gastrocnemius, and soleus, were collected on days 0 (no injury), 1, 3, and 7 following HLI surgery, and single-cell suspensions were generated using mechanical dissociation followed by enzymatic digestion with 0.05% Pronase (Sigma, 537088) for 1 h. Cells were vacuum filtered as described above and subsequently stained with PI, anti-CD45-Alexa Fluor 488 (clone HI30, Invitrogen, MHCD4520), and FITC anti-CD31 (BioLegend, 102506). Using FACS, 150,000 PI- cells and an additional 150,000 PI-/CD31-/CD45- cells were isolated. The latter population (“depleted” cells) was isolated to increase the representation of non-hematopoietic and non-endothelial cells, given the relative scarcity of muscle stem cells and fibro-adipogenic progenitors. Live cells and “depleted” cells were pooled at a 1:1 ratio and a total of 16,000–24,000 cells used for scRNA sequencing.

### Single-cell RNA sequencing library generation

Cells were collected for single-cell RNA-seq analysis as described above. Single-cell RNA-seq libraries were generated using Chromium Next GEM Single Cell 3’ Reagent Kits v3.1 (10x Genomics) according to the manufacturer’s protocol.

### Human muscle immunofluorescent staining

Human skeletal muscle samples from both ischemic (distal) and non-ischemic (proximal) muscle were obtained from surgical amputation specimens. For AKAs, the proximal muscle specimen was obtained from the vastus medialis and the distal specimen from the tibialis anterior. For BKAs, the proximal and distal specimens were both obtained from the tibialis anterior. Tissue was harvested and embedded in OCT compound using liquid nitrogen; 8-μm sections were prepared on microscope slides using cryostat sectioning for histological analysis. Frozen sections were allowed to come to room temperature and fixed in 4% paraformaldehyde for 10 min, permeabilized with 0.1% Triton X-100 in PBS for 5 min, and then washed in PBS. Blocking solution (5% normal donkey serum in PBS) was applied for 30 min at room temperature followed by primary antibody staining overnight at 4°C using anti-CD206 (R&D systems, AF2534), anti-CD11b (Cell Sciences, MON1019-1, clone Bear-1), and anti-dystrophin (Thermo Scientific, RB-9024-P). Tissue sections were then washed using PBS, incubated with the secondary antibody (ThermoFisher Scientific) for 1 h at room temperature, and counterstained with 1ug/mL Hoechst 33342 (Thermo Scientific, 6629) for 5 min at room temperature. The tissue sections were mounted with Fluoromount-G™ Mounting Medium (ThermoFischer Scientific, 00-4958-02). Images were acquired using a Zeiss Axio Imager Z2 Upright Microscope at x200 magnification.

### Mouse muscle immunohistochemistry

The entire tibialis anterior muscle was obtained. Immediately upon harvest, muscle tissue was harvested and embedded in optimal cutting temperature compound (“OCT,” Tissue Tek, 4583) using liquid nitrogen; 8-mm sections were prepared on microscope slides using a cryostat sectioning and were stored at −80°C. Frozen sections were allowed to come to room temperature, fixed in 4% paraformaldehyde in phosphate buffered saline (PBS) for 10 min, washed in PBS, and then permeabilized with 0.3% Triton X-100 in phosphate buffered saline (PBS) for 10 min. Antigen retrieval was performed on samples stained with Pax7 by incubation for 10 min in 10 mM sodium citrate pH 6.0 in a pressure cooker. Mouse samples were blocked for 30 min with 20 mg/ml goat anti-mouse IgG (Jackson Immunoresearch, 115-007-003) in 0.1% Triton X-100/PBS (PBST)/1% bovine serum albumin, followed by 30 min with 5% normal goat serum (Thermo Fisher, 16210064). Primary antibody staining was conducted overnight at 4°C using anti-dystrophin (Thermo Fisher, RB-9024-P), anti-Pax7 (Developmental Studies Hybridoma Bank, Pax7), anti-Myh3-Alexa Fluor 594 (Santa Cruz, sc-53091 AF594), anti-CD31 (Biolegend, 160202), and anti-laminin (Sigma, L9393). Pax7 samples were then stained using an Alexa Fluor 594 tyramide amplification kit (Thermo Fisher, B40935) according to the manufacturer’s instructions. Other tissue samples were washed using PBST, incubated with 1:500 dilutions of secondary antibodies (Thermo Fisher, A-11020, A-11070, A-11007) and 1ug/mL Hoechst 33342 (Thermo Fisher, 62249) in 5% goat serum/PBST for 1 h at room temperature. The tissue sections were washed with PBST and mounted with Fluoromount-GTM Mounting Medium (Thermo Fisher, 00-4958-02). Images were acquired using a Zeiss Axio Imager Z2 Upright Microscope at ×100 and ×200 magnification. Pax7-positive nuclei were scored manually using Zeiss Zen Microscopy software. CD31-positive cells and Myh3-positive fibers were scored using ImageJ.

### Macrophage isolation using FACS

Hind limb muscles were collected from C57BL/6 and BALB/c mice on post-operative day 3 following HLI surgery. Single-cell suspensions were generated as described above. Cells were blocked with purified anti-mouse CD16/32 antibody (Biolegend, #101301) for 10 min. Primary antibody staining was performed using anti-CD45-Alexa Fluor 488 (clone HI30, Invitrogen, MHCD4520), anti-CD11b (clone M1/70, Invitrogen, 12-0112-81), and anti-F4/80-biotin (clone A3-1, Bio-rad, MCA497BT) antibodies for 40 min. Streptavidin-PE/Cy7 (Biolegend, 405206) was used as a secondary reagent for anti-F4/80-biotin (PMID: 25896247). Using FACS, macrophages were isolated by PI-/CD45+/CD11b+/F480+ gating.

### Bulk RNA sequencing

Total RNA was extracted using TRIzol Reagent (Invitrogen) according to the manufacturer's protocol. First-strand reverse transcription and template switching was performed using an Oligo(dT) primer (dT30VN-ME-A), a locked nucleic acid-containing TSO (NotI-TSO), and Superscript IV reverse transcriptase (Invitrogen, # 18090050). PCR preamplification of cDNA was performed with IS PCR and Tn5ME-A-aHic using 2X KAPA PCR mix (Kapa Biosystem, KK2602) followed by cleanup using SPRISelect beads (Beckman Coulter, REF B23319). DNA was digested by NotI-HF (NEB, #R3189L), subjected to tagmentation using Tn5 assembled with adaptors Tn5ME-A/Tn5MErev and Tn5ME-B/Tn5MErev. A Zymo DNA clean and concentrator kit (Zymo, R1014 ) was utilized [32]. Library PCR was performed using unique combinations of Nextera-PCR i5/i7 primers. DNA strands between 400 and 600 bp were selected by gel extraction using Zymoclean Gel DNA recovery kit (Zymo, #D4002). DNA libraries were submitted for next-generation sequencing using paired-end sequencing.

### Cell proliferation assay

Skeletal muscle stem cells were isolated from uninjured young adult C57BL/6 and BALB/c hindlimb skeletal muscle and myogenic cells were expanded in culture using an established protocol [33]. Cells were seeded in 96-well plates and 24 h after plating treated with: THBS1 (R&D systems, 7859-TH-050), syndecan-4 Ab (BD Pharmingen, 550350), normal rat IgG2a (EMD Millipore, MABF 1077Z), IGF-1 (R&D systems, 791-MG), FGF2 (Thermo Fisher, PHG0367), or vehicle (PBS) for 24 h. After 72 h of culture with the indicated ligands, EdU was added to the medium, the cells were cultured for an additional 6 h, and then fixed. Cells were analyzed using the Click-iT EdU Cell Proliferation Kit for Imaging (Invitrogen, C10337) according to the manufacturer’s instructions.

### Mouse scRNA-seq data processing and analysis

The sequencing reads (10× Genomics) were processed using the Cell Ranger pipeline (v3.1.0,) with GRCm38 reference genome. The output filtered matrices of different samples as input files for downstream analyses using the R package Seurat (v4.0.1) [34]. Genes expressed by less than three cells were removed. Cells with unique feature counts over 4100, under 1000, or greater than 25% mitochondrial RNA counts were filtered. Meanwhile, cells that were recognized as doublets by the Python package Scrublet (v0.2.3) were removed. The different Seurat objects from different samples were combined to create a new object.

The gene expression levels of the combined object for each cell were normalized and log-transformed by the NormalizeData function. Scaled data of all cells by ScaleData function was used for principal component analysis based on 2000 highly variable genes with RunPCA function. The harmony algorithm was then used for batch correction [35]. The resulting Harmony embeddings, instead of PCA, were used in non-linear dimensional reduction and nearest neighbor graph construction. The clusters were defined using the FindClusters function (resolution = 0.6) and marker genes for each cluster were identified by running the FindAllMarkers function. Cell type annotations of each cluster were made based on these marker genes. These steps were also used for the mouse macrophage sub-clusters analysis.

The satellite cells were exacted from the Seurat object of each sample for normalization using sctransform and then merged as a new object. The SelectIntegrationFeatures function was used to choose the top scoring features for the satellite cell integrated object. Then the UMAP and sub-clusters were defined using the steps described above (RunPCA, RunHarmony, RunUMAP, FindNeighbors, FindClusters). The differentially expressed genes (DEGs) were identified using the FindMarkers function. The trajectory inference and pseudotime calculations on the integrated object were performed using Monocle3 [36]. In detail, the Seurat object was converted to Monocle3 “cell_data_set” objects. Then the cluster_cells and learn_graph functions were used to cluster cells with parameter “k=25” and build the trajectory graph with parameter “rann.k=45”, sequentially. The order_cells function was used to calculate where each cell falls in pseudotime with parameter “root_pr_nodes = ‘Y_4’”. Cell–cell communication between macrophages and satellite cells was inferred using CellphoneDB v3.1 [37–39]. The AddModuleScore function was used to calculate module scores for feature expression programs related to the inflammatory response in macrophages based on homologous genes from the “GO:0006954~inflammatory response” term (Additional file 3: Table S2). The gene sets for the macrophage GSEA analysis were downloaded from MSigDB [40–42].

### Human scRNA-seq data processing and analysis

The sequencing reads from CLTI patient samples were processed using the Cell Ranger pipeline (v3.1.0,) with GRCh38 reference genome. The output filtered matrices of each sample were used as input files for downstream analyses performed using the R package Seurat (v4.0.1) [34]. The same cell filtering criteria and processing steps used in the mouse scRNA-seq data analysis were used to analyze the merged Seurat object of patient samples using the functions NormalizeData, FindVariableFeatures, ScaleData, RunPCA, RunHarmony, RunUMAP, FindNeighbors, and FindClusters (resolution = 0.5), sequentially. The FindMarkers function was used to identify marker genes of each cluster and DEGs between different groups. The raw scRNA-seq data from healthy human skeletal muscle samples were downloaded from GSE143704. The same methods used to process the CLTI patient sample data were used to process these published data. The Seurat objects of patient and healthy muscle samples were merged together to find clusters (resolution = 0.8) using the steps described above. Cell–cell communication between macrophages and MuSCs was inferred using CellChat v1.4 [39] and CellphoneDB v3.1 [37, 38], separately.

### Bulk RNA-seq data analysis

Bulk RNA-seq reads were aligned to the GRCm38 reference genome by STAR v2.7.4a with “--sjdbOverhang 99” [43]. FeatureCounts v1.6.3 was used to determine the read counts for each gene [44]. The DEGs were identified using DESeq2 [45] v1.30.1 with a threshold of adjusted *p*-value less than 0.05 and fold change greater than 2. GO enrichment analysis was performed with these DEGs via DAVID [46, 47]. The bigwig files generated by bamCoverage v3.5.1 [48] with RPKM normalization from alignment of reads (bam files) were used for read coverage visualization in the Integrative Genomics Viewer (IGV v 2.11.9) [49].

## Results

### The ischemic-injured muscle in CLTI patients is enriched with pro-inflammatory macrophages

**Fig. 1:**
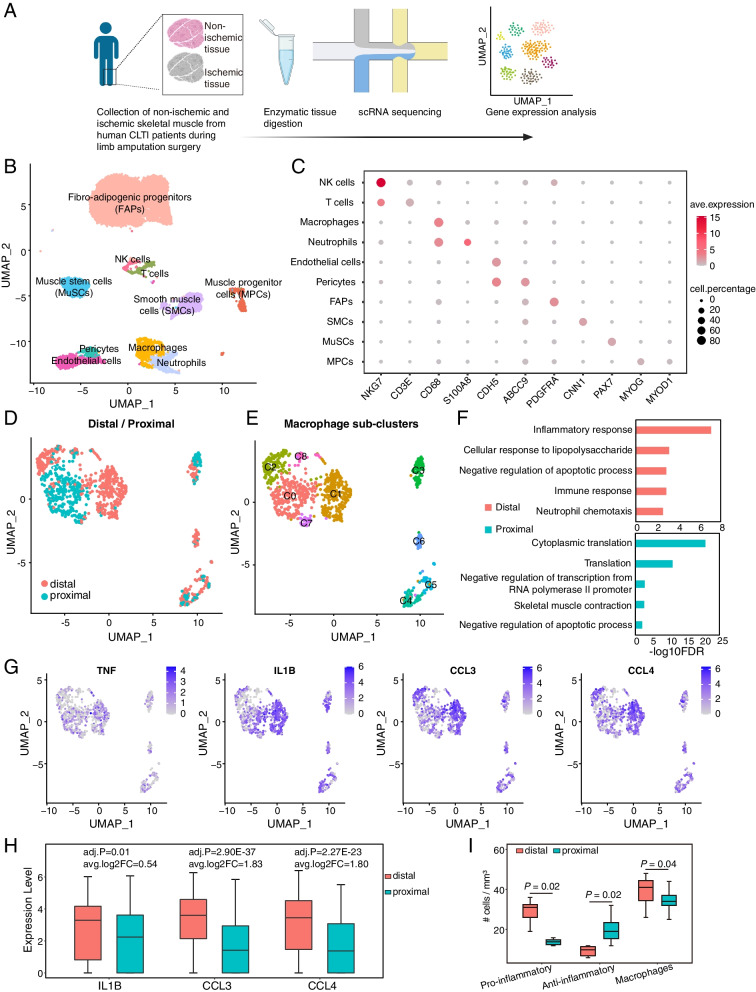
Single-cell transcriptional profiling of human CLTI patients’ limb muscle in non-ischemic versus ischemic conditions. **A** Schematic diagram illustrating the generation of scRNA-seq datasets using proximal and distal tissue from human CLTI skeletal muscle. **B** Uniform manifold approximation projection (UMAP) visualization showing cell populations (*n* = 16,201) from non-ischemic and ischemic tissues of CLTI patients (*n* = 3 donors, paired proximal and distal tissues were analyzed). **C** Dot plot displaying the expression of marker genes for each cell population. Dot size represents the percentage of cells that positively detect the transcripts, and the color scale indicates average expression levels. **D**, **E** UMAP visualization of macrophages in proximal (blue, non-ischemic) and distal (pink, ischemic) skeletal muscle (**D**) and sub-clusters (**E**, C0-C8). **F** Top five Gene Ontology (GO) terms enriched by differentially expressed genes (*P*-value < 0.05 & |log2FoldChange| > 0.25) between distal (pink, ischemic) clusters (1 and 2) and proximal (blue, non-ischemic) cluster (0). Pink and blue bars represent the GO terms enriched in distal and proximal conditions, respectively. **G** Feature plots showing the expression of pro-inflammatory genes in macrophages. **H** Quantification of representative pro-inflammatory gene expression in proximal versus distal macrophages. Adjusted *p*-values (adj.P) and log fold changes of average expression (avg.log2FC) were calculated by Wilcoxon rank-sum test. **I** Quantification of CD11b+/CD206+ and CD11b+/CD206- macrophages in ischemic and non-ischemic CLTI patient muscle specimens. *P* values were calculated by paired Wilcoxon rank-sum test

To understand the pathological changes in ischemia-injured skeletal muscle, we carried out single-cell RNA-seq analysis of fresh skeletal muscle samples from CLTI patients undergoing limb amputation surgery. The biopsies were taken from both the distal (ischemic) and proximal (non-ischemic) regions of the amputated limb and dissociated into single cell suspensions. It should be noted that amputations occur at a level where the tissue is healthy and normoxic to ensure wound healing. Hence, the proximal tissues are not ischemic. The live cells were FACS sorted and immediately subjected to single-cell transcriptome analysis (Fig 1A). Importantly, obtaining matched proximal and distal tissue from the same individual allowed us to examine the specific pathological changes caused by chronic ischemia while controlling for differences in the genetic background and health conditions of each patient. Using pairs of matched proximal and distal muscle tissues from three representative CLTI patients (Additional file 1: Fig S1A, bottom; Table 1), we recovered a total of 16,201 high-quality cells for downstream bioinformatics analysis. After correcting for batch effect and patient-specific biases (detailed in Materials and Methods), all cells were uniformly dispersed throughout the UMAP space (Additional file 1: Fig S1B). We next analyzed cells based on the anatomic location of the samples from which they were obtained in the amputated limb (distal, ischemic; proximal, non-ischemic) to determine if there was a hypoxic transcriptional signature in cells from the distal tissue. Indeed, the cells from the distal limb displayed increased expression of HIF1A compared to those from the proximal limb (Additional file 1: Fig S1C). This finding, in concert with our computed tomography (CT) scans for each patient (Additional file 1: Fig S1A, bottom), demonstrates that the distal tissue specimens are ischemic compared to the proximal specimens.

**Table 1:** Human CLTI patient demographics (*N* = 10)

Patients (*N*)	Gender (M/F)	Age (mean ± SD)	BMI (mean ± SD)	Diabetes (Y/N)	HTN (Y/N)	HLD (Y/N)	CKD (Y/N)	CAD (Y/N)	ABI	Analysis
3	2/1	80 ± 8.19	30.13 ± 5.85	1/2	3/0	3/0	1/2	1/2	n/a: 3	scRNA-seq analysis
7	7/0	66 ± 10.93	23.36 ± 4.10	5/2	7/0	6/1	5/2	7/0	0.4 - 0.9: 2< 0.4: 2 NC: 2 n/a: 1	Histologic analysis

*ABI* Ankle brachial index, *BMI* Body mass index, *CAD* Coronary artery disease, *CKD* Chronic kidney disease, *HLD* Hyperlipidemia, *HTN* Hypertension, *n/a* Not available, *NC* Non-compressible

The 16,201 cells were annotated into ten major cell types, including fibro-adipogenic progenitor cells (FAPs), muscle stem cells (MuSCs), muscle progenitor cells (MPCs), endothelial cells, macrophages, neutrophils, T cells, pericytes, NK cells, and smooth muscle cells (SMCs) based on the expression of well-defined cell type-specific marker genes (Fig. 1B, C; Additional file 1: Fig S1D, S1E). We further analyzed the proportional distribution of each cell type in both proximal and distal conditions from the three CLTI patients. This analysis indicates a decrease in the proportion of endothelial cells, pericytes, T cells, MuSCs, and MPCs, and an increase in the presence of FAPs and neutrophils in the distal tissues compared to the proximal non-ischemic tissues (Additional file 1: Fig S1F). Our single-cell atlas of CLTI patient samples, encompassing matched proximal and distal tissue samples, suggests that chronic ischemic damage alters the cellular landscape of skeletal muscle in CLTI patients.

Intriguingly, when macrophages were segregated into non-overlapping populations on a new UMAP space with increased resolution, significant differences were seen between cells derived from proximal versus distal tissue (Fig. 1D). The macrophages were separated into nine sub-clusters (Fig. 1E). Of these, cluster 0 was composed primarily of cells from non-ischemic tissue, while clusters 1 and 2 were predominantly composed of macrophages from ischemic-injured distal tissue (Fig. 1E). We further identified genes differentially expressed in clusters 1 and 2 versus cluster 0 (Wilcoxon test, *p*-value < 0.05, log2 fold change > 0.25, Additional file 3: Table S2) and found that the genes highly expressed in clusters 1 and 2 were enriched for Gene Ontology (GO) terms related to pro-inflammatory pathways (Fig. 1F). Several well characterized pro-inflammatory genes, such as *TNF*, *IL1B*, *CCL3*, and *CCL4* [50], as well as the key hypoxia-responsive gene *HIF1A* [51], were expressed at significantly higher levels in macrophages from distal tissue versus those from proximal muscle (Fig. 1G, H; Additional file 1: Fig S1G). These results demonstrate that macrophages in the distal tissues of CLTI patients are impacted by chronic ischemic-injury and display a pro-inflammatory phenotype.

To experimentally validate these findings, we collected distal and proximal skeletal muscle samples from another seven CLTI patients (Table 1) and immunostained the tissue sections with antibodies against the pan-macrophage marker CD11b and the anti-inflammatory macrophage marker CD206 (Additional file 1: Fig S1H, S1I). According to previously published criteria [52, 53], we designated the CD11b+/CD206+ cells as anti-inflammatory macrophages and the CD11b+/CD206- cells as inflammatory macrophages. In the ischemic-injured distal muscle, we found on average 1.97-fold more pro-inflammatory macrophages compared to the non-ischemic condition (Fig. 1I, *p*-value = 0.02, paired samples Wilcoxon test). In contrast, there were on average 2.07-fold more anti-inflammatory macrophages in the non-ischemic proximal muscles (Fig. 1I, I-value = 0.02, paired samples Wilcoxon test). Considering all these results, we conclude that pro-inflammatory macrophages are indeed enriched in the ischemic-damaged limb muscle of CLTI patients.

### Single-cell transcriptome analysis of regenerative versus CLTI-like mouse limb muscle following hind-limb ischemia surgery

**Fig. 2:**
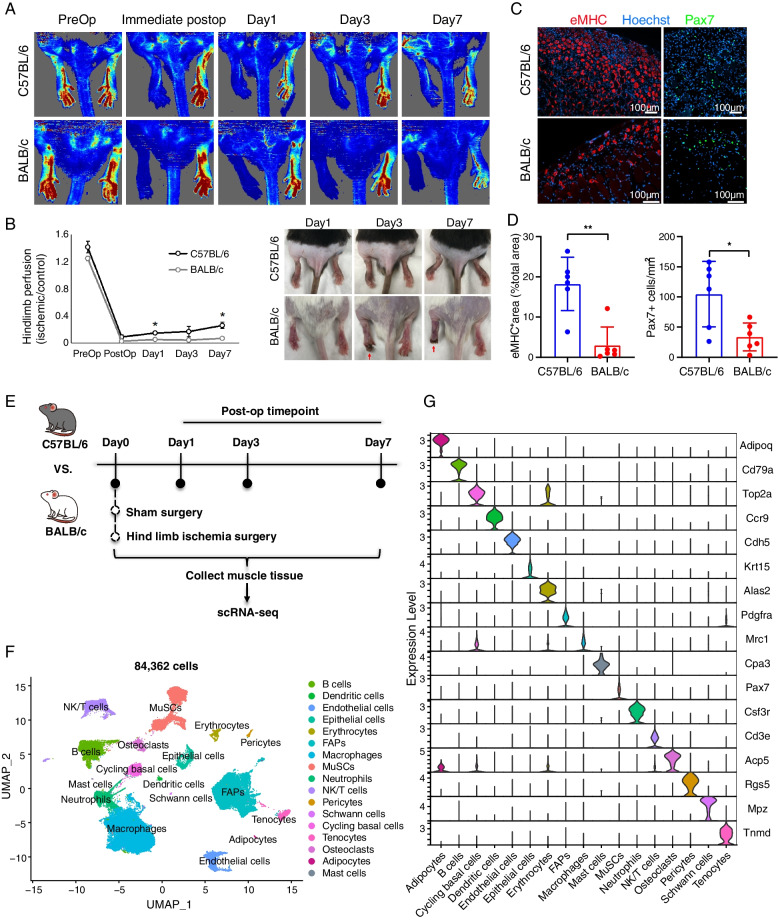
Single-cell RNA-seq analysis of hind limb ischemia (HLI) surgery induced limb muscle regeneration and damage responses in C57BL/6 and BALB/c mice. **A** Perfusion imaging of C57BL/6 (top) and BALB/c (bottom) mouse strains before and after HLI surgery (Pre-operatively and Post-operatively, respectively) and on post-op days 1, 3, and 7. **B** (Left) Quantification of limb perfusion as determined by perfusion imaging at indicated timepoints. Left hindlimb (HLI surgery) perfusion normalized to right hindlimb perfusion for each mouse. *n* = 3 per mouse strain, per timepoint. *p < 0.01. (Right) Representative images of mice on post-op days 1, 3, and 7 following HLI surgery. Red arrow indicates ischemic changes apparent on post-op days 3 and 7 in BALB/c mice. **C** Representative immunofluorescence staining images of mice on post-op day 7 following HLI surgery. eMHC (red) indicates regenerated muscle fibers; Pax7 (green) indicates satellite cells. **D** Quantification of eMHC+ area (left panel) and Pax7+ cell counts (right panel) shown in **C**. *n* = 6 mice per group. Data expressed as mean ± SEM. *p ≤ 0.05, **p ≤ 0.01. **E** Experimental design of mice scRNA-seq analysis of mouse models of HLI (*n* = 2 per mouse strain, per timepoint). **F** UMAP visualization of the scRNA-seq atlas assembled from all samples and time points. **G** The expression of cell type marker genes used for each cell type/cluster annotated in **F**

Next, we sought to determine whether the change of the inflammatory response of ischemic limb muscle is associated with the tissue loss in CLTI. Since it is not feasible to obtain clinical samples from CLTI patients through disease progression over time, we employed a murine model of CLTI in which hind limb ischemia (HLI) surgery is used to ligate the femoral artery in BALB/c and C57BL/6 mice to assess the temporal dynamics of limb tissue loss in CLTI (Fig. 2A, B). As noted, following HLI surgery, BALB/c mice develop a CLTI-like, profound tissue loss phenotype and paw necrosis, and a significant reduction of Cd31+ endothelial cells as assessed by capillary density analysis compared to C57BL/6 mice (Fig. 2B, right; Additional file 1: Fig S2A) [13, 19, 54]. In contrast, C57BL/6 mice display very minor, if any, tissue loss, despite experiencing a similar 80–90% reduction in limb blood flow after HLI (Fig. 2B). Consistent with previous results [13], the ischemic tibialis anterior (TA) muscle of C57BL/6 mice exhibited a potent muscle regenerative response following HLI, indicated by 6.2-fold greater expression of embryonic myosin heavy chain (eMHC) and 3.1-fold more Pax7+ satellite cells compared to BALB/c mice at 7 days post-injury (dpi) (Fig. 2C, D; Additional file 1: Fig S2B). Therefore, BALB/c mice represent a murine model of CLTI with permanent tissue loss, whereas C57BL/6 mice are resistant to ischemia-induced muscle damage and display a potent skeletal muscle regenerative program following HLI.

Next, we performed scRNA-seq analysis utilizing cells collected from hindlimb muscles, including tibialis anterior (TA), gastrocnemius, and soleus, from BALB/c and C57BL/6 mice, following HLI surgery, at intervals ranging from 1 to 7 days post-injury (dpi). Cells obtained from the unligated limb, following sham surgery, served as the control group. We incorporated two biological replicates for each experimental condition (Fig. 2E). In total, we recovered 84,362 high-quality single cells from the two mouse strains at four-times (Fig. 2F, Additional file 1: Fig S2C). We identified 17 major cell types, including MuSC/MPCs, immune cells, and FAPs (Fig. 2F). These annotated cell types express high levels of expected marker genes that were defined in previous studies of scRNA-seq analysis of mouse skeletal muscle regeneration (Fig. 2G, Additional file 1: Fig S2D). Notably, a marked increase in HIF1A expression was observed in cells harvested from post-HLI limbs in both mouse strains, relative to cells from non-ischemic limbs, thereby demonstrating the ischemic impact on cells following HLI (Additional file 1: Fig S2E). These scRNA-seq datasets thus provide the first reference atlas to examine the temporal dynamics of cell populations and their gene expression patterns in mouse strains that display either effective or failed skeletal muscle regeneration following limb ischemia.

### Pro-inflammatory macrophages are enriched in the ischemic-damaged limb muscle of mice subjected to HLI

**Fig. 3:**
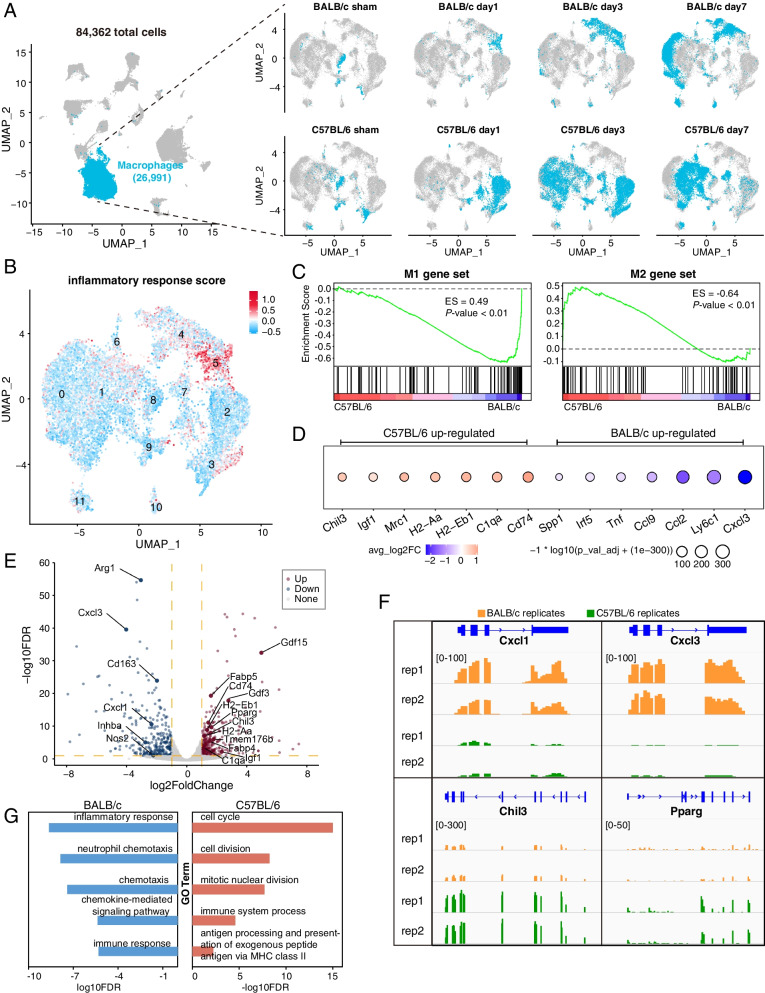
scRNA-seq analyses reveals that the pro-inflammatory macrophages are enriched in the BALB/c limb following HLI surgery. **A** UMAP plots illustrating the distribution of macrophages (depicted in blue) and other cells (depicted in grey) from C57BL/6 and BALB/c mice at specified time intervals. The left UMAP plot aggregates all cells from the two strains across all time points. In the right-hand UMAP plots, macrophages for the distinct time points and individual strains are highlighted in blue, while all remaining cells are represented in grey. **B** The inflammatory gene module score is high in cluster 5 cells, which are specific to BALB/c mice. Red color indicates high gene module score. **C** GSEA enrichment analysis reveals that M1 macrophage markers are highly expressed in BALB/c macrophages, while M2 macrophage markers are highly expressed in C57BL/6 macrophages. Macrophage gene expression patterns are assessed using scRNA-seq data. **D** Dot plot showing differentially expressed genes in macrophages between C57BL/6 and BALB/c mice on day 3 post-ischemia. Dot size represents -log10 FDR; color scale indicates log2-fold change in gene expression. Pink and blue colors indicate genes upregulated in C57BL/6 and BALB/c, respectively. **E** Volcano plot displaying differentially expressed genes from bulk RNA-seq analysis of macrophages purified from BALB/c and C57BL/6 mice at 3 days post-HLI. Red and blue dots represent upregulated genes in C57BL/6 and BALB/c mice, respectively. **F**: Genome browser snapshots showcasing bulk RNA-seq data at specified gene loci in macrophages derived from both strains on day 3 post-injury. Two biological replicates (rep1, rep2) are included for each condition. **G** Top five GO terms enriched by differentially expressed genes (FDR < 0.05, log2FoldChange>1) from panel **E**. Red and blue bars correspond to GO terms enriched in C57BL/6 and BALB/c mice, respectively

We next explored whether differences exist in the macrophage populations in the ischemic hindlimbs of C57BL/6 and BALB/c mice. Fine resolution sub-clustering analysis of a total of 26,991 macrophages revealed 12 sub-clusters (Additional file 1: Fig S3A, clusters 0-11), which display temporal-, strain-, and cluster-specific gene expression patterns (Fig. 3A, Additional file 1: Fig S3B, Additional file 4: Table S3). Notably, clusters 4 and 5 were dominated by BALB/c cells, while clusters 1, 2, 3, 7, 8, and 9 were made up primarily of C57BL/6 macrophages (Fig. 3A, right). At 3 dpi, the macrophages from BALB/c and C57BL/6 mice were segregated into two non-overlapping populations on the UMAP (Fig. 3A**,** right, day 3), indicating drastically different macrophage gene expression programs at day 3 in the two strains. Significantly, the BALB/c-specific cluster 5 displayed a strong pro-inflammatory gene expression signature, as demonstrated by a high inflammatory response score, which was computed based on the expression levels of genes associated with the GO term “inflammatory response” (Fig. 3B, Additional file 3: Table S2, detailed in material and methods). Macrophages can be classified largely into the pro-inflammatory M1 and anti-inflammatory/pro-regenerative M2 states based on their in vitro phenotype in response to inflammation stimulation [55]. Using this convention, we found that anti-inflammatory M2 genes were highly expressed in the C57BL/6 macrophages, while pro-inflammatory M1 genes were highly expressed in macrophages from BALB/c mice (Fig. 3C, D). These results suggest that following HLI, the non-regenerative BALB/c limb muscles are enriched with macrophages exhibiting a pro-inflammatory phenotype compared to those in the regenerative C57BL/6 muscle.

To experimentally validate the findings from scRNA-seq analyses, we purified CD11b+/F4/80+ macrophages by FACS from the hindlimb muscle of C57BL/6 and BALB/c mice at 3 dpi following HLI for bulk RNA-seq analysis (Additional file 1: Fig S3C). We identified 289 down-regulated and 320 up-regulated genes in C57BL/6 versus BALB/c macrophages (Fig 3E, Additional file 5: Table S4, DEseq2, fold change > 2, FDR < 0.05). Many pro-inflammatory genes, such as *Arg1*, *Cxcl3*, and *Cxcl1* [30], were highly expressed in the BALB/c macrophages (Fig. 3E, F). In contrast, anti-inflammatory and pro-regenerative genes, such as *Chil3*, *Igf1*, Gdf15, and *Gdf3* [56, 57], were highly expressed in C57BL/6 3 dpi macrophages (Fig. 3E, F; Additional file 1: Fig S3D). The genes highly expressed in 3 dpi BALB/c macrophages were enriched for GO terms associated with inflammatory response, chemotaxis, and immune response (Fig. 3G, Additional file 5: Table S4). These results collectively highlight the clear transcriptional differences in macrophages present in the regenerative (C57BL/6) and non-regenerative (BALB/c) limbs following HLI and demonstrate that a strong pro-inflammatory transcriptional signature of macrophages, particularly at 3 dpi, is associated with the limb tissue loss phenotype of BALB/c mice.

### Impaired muscle regeneration in BALB/c ischemic limbs characterized by premature myogenic differentiation and proliferative deficit of MuSCs

**Fig. 4:**
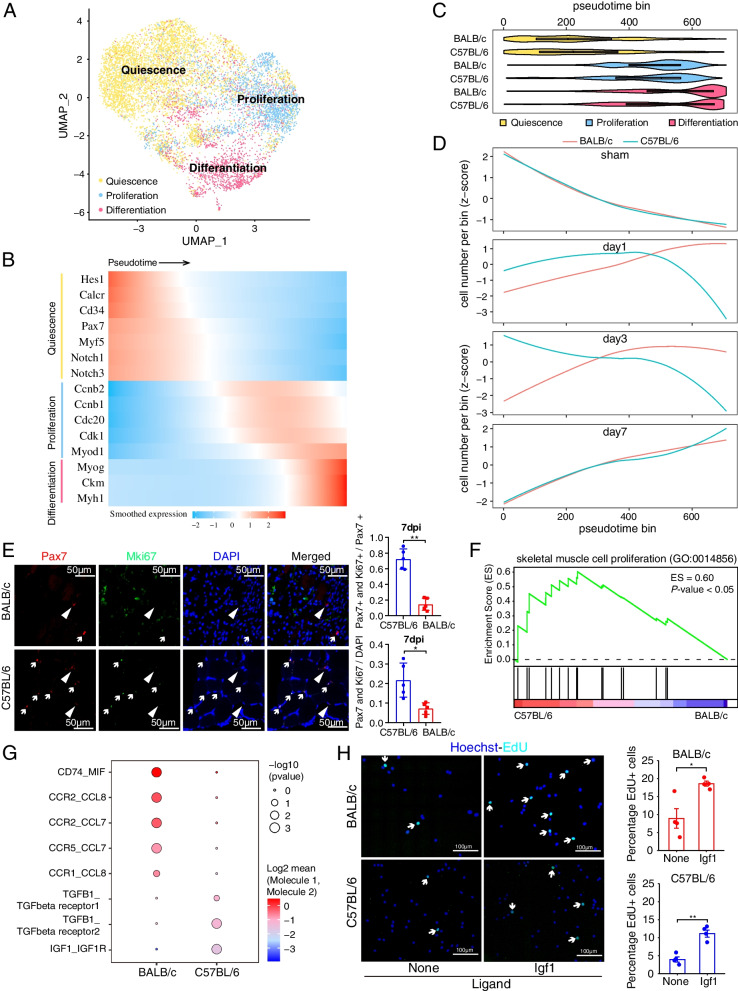
MuSCs/MPCs in BALB/c mice undergo precocious differentiation after HLI surgery. **A** UMAP representation displaying quiescent (depicted in yellow), activated/proliferative (in blue), and differentiating (in pink) muscle stem cells (MuSCs) and muscle precursor cells (MPCs). Total MuSC/MPC count stands at 9,217. **B** MuSCs/MPCs from part **A** are arranged in a pseudotime sequence, initiating from quiescent cells (left) and advancing to activated/proliferative (middle) and subsequently to differentiating cells (right). The heatmap's color gradient represents the expression intensity of the specified genes in MuSCs/MPCs in accordance with the pseudotime progression. **C** (Top) Violin plot showing the distribution of quiescent (depicted in yellow), activated/proliferative (in blue), and differentiating (in pink) MuSCs/MPCs in the two strains along the pseudotime trajectory shown in **B**. (Bottom) Curve plot showing the distribution of MuSC/MPCs at indicated time points, before (sham) and 1, 3, and 7 dpi post-HLI, in the two mouse strains along the pseudotime trajectory shown in **B**. **D** Gene set enrichment analysis (GSEA) highlighting the genes related to “skeletal muscle cell proliferation” are significantly enriched in the up-regulated genes in MuSCs/MPCs in C57BL/6 mice compared to those in BALB/c mice. **E, F** At 7 dpi, TA muscles were collected from both C57BL/6 and BALB/c for immunostaining using antibodies against Pax7 and cell proliferation marker Mki67. **E** *n* = 5 mice per strain. Data expressed as mean ± SEM. *p ≤ 0.05, **p ≤ 0.01. **F** Representative Pax7 (red) and Mki67 (green) immunofluorescence staining images along with DAPI (blue). **G** Representative RNAscope data showing that Adgre1+ macrophages (F4/80, green) and Myod1+ MuSC/MPCs (red) are spatially proximal to each other in the limb muscle of BALB/c and C57BL/6 mice at day 3 after HLI. Three mice per strain were used for RNAscope analysis. **H** Inferred ligand-receptor interactions between macrophages and MuSCs in BALB/c and C57BL/6 following HLI at 3 days post HLI surgery. **I** IGF1 promotes proliferation of primary MPCs purified from BALB/c and C57BL/6 strains. (Left) Representative images of EdU incorporation by C57BL/6 and BALB/c primary MPCs cultured with or without recombinant IGF1 for 72 h. EdU was added to the culture medium 6 h prior to cell fixation and imaging. Nuclei stained with Hoechst. Arrows indicate EdU+ cells. (Right) Quantification of the percentage of EdU+ cells for the indicated strains and treatment conditions. *P < 0.05, **P < 0.005

MuSCs directly contribute to muscle regeneration through their activation from the initial quiescent state and subsequent proliferation, differentiation, and fusion [58]. To delineate strain-specific responses of MuSCs and MuSC-derived muscle precursor cells (MPCs) following HLI, we performed an in-depth analysis of all the scRNA-seq data on *Pax7*+ MuSCs and *Myod*+ MPCs. First, the MuSCs/MPCs were classified into quiescent (*Pax7* high), activated/proliferating (*MyoD*, *Ki67* high), early-differentiating (*MyoG*+), and late-differentiating (*Ckm*+) states (Fig. 4A, Additional file 1: Fig S4A). Next, we conducted pseudotime trajectory analysis to rank the MuSCs/MPCs based on their transcriptome similarities (Additional file 1: Fig S4B). This approach showed that MuSCs/MPCs ranked in the early part of the pseudotime trajectory expressed high levels of quiescence marker genes (*Hes1*, *Calcr*, *Cd34*, *Pax7*, *Myf5*, *Notch1/3*), while cells ranked later in the pseudotime trajectory expressed high levels of the activation marker gene *MyoD* and cell cycle-related genes (*Cdnb1/2*, *Cdc20*, *Cdk1*) or high levels of marker genes associated with myogenic differentiation (*MyoG*, *Ckm*, *Myh1*). Therefore, the MuSCs/MPCs in our dataset, when ranked along this pseudotime trajectory, exhibit the expected cellular states, ranging from quiescence to activation/proliferation and differentiation (Fig. 4B). Significantly, along the pseudotime trajectory, we noted that MuSCs/MPCs from both BALB/c and C57BL/6 mice begin at the initial quiescent state (Fig. 4C, top) and transition into the activation/proliferation and differentiation states (Fig. 4C, middle and bottom), demonstrating that MuSCs/MPCs from the BALB/c strain do not intrinsically lack regenerative capacity.

It is well established that the MuSCs/MPCs proliferation phase is critical for efficient muscle regeneration as it is required to produce sufficient MPCs for myogenic differentiation and fusion and that the timing of the transition from the proliferative state to the differentiation state is also important [59]. We found that before HLI (sham) and at 7 dpi, the MuSCs/MPCs collected from the two mouse strains were well-aligned on the pseudotime trajectory and mirrored each other closely in both the early (quiescent) and late (differentiation) stages of pseudotime (Fig. 4D, sham and day 7). In contrast, the MuSCs/MPCs exhibited drastic strain-specific differences at 1 dpi and 3 dpi (Fig. 4D, day 1 and day 3). At both timepoints, a larger fraction of C57BL/6 MuSCs/MPCs (blue lines) were in the activation/proliferation phase, whereas the vast majority of the BALB/c cells (red lines) occupied the late, differentiation stage of the pseudotime trajectory (Fig. 4D, day 1 and day 3). This pattern suggests that the BALB/c MPCs committed to differentiation prematurely without sufficient proliferation. Indeed, the genes highly expressed in C57BL/6 compared to BALB/c MPCs at 3 dpi were significantly enriched for GO terms “skeletal muscle cell proliferation” (Fig. 4F). Finally, through immunofluorescence analysis of post-HLI limb muscle, we found that in BALB/c mice the proportion of proliferative Ki67/Pax7-double positive MuSCs was substantially less than in C57BL/6 mice (Fig. 4E). Collectively, these results suggest that the failure of skeletal muscle regeneration in the BALB/c model of CLTI is at least in part due to inadequate proliferation and premature differentiation of MuSCs/MPCs.

### The pro-inflammatory niche is associated with premature differentiation of MuSCs in BALB/c mice following HLI

MuSC regenerative events are substantively coordinated and supported by macrophage-derived ligands [28, 60, 61]. To address whether the inflammatory macrophages in BALB/c muscle are associated with premature differentiation of MuSCs following HLI, we sought to identify candidate ligand-receptor pairs to account for the disparate, strain-specific macrophage-MuSC cross-talk. We assessed the probability of intercellular communication between macrophages and MuSCs using a computational method called CellPhoneDB [37]. We found that at 3 dpi, C57BL/6 MuSCs are likely responsive to well-characterized pro-regenerative cytokines secreted by macrophages, such as TGFB1 and IGF1 (Fig. 4G), which play critical roles in promoting MPC proliferation and preventing their premature differentiation [56, 62, 63]. In contrast, TGFB1 and IGF1 pathways were not detected in BALB/c mice in macrophage-MuSC intercellular communication (Fig. 4G). Notably, at 3 dpi, the IGF1 receptor (*Igf1r*) is specifically expressed in C57BL/6 MuSCs/MPCs (Additional file 1: Fig S4E) and the expression of *Igf1* in C57BL/6 macrophages is elevated compared to BALB/c macrophages at the same timepoint (Additional file 1: Fig S3D). This data suggests that signaling communication, specifically within the IGF1 pathway, between macrophages and MuSC/MPCs plays a pivotal role in fostering MuSC/MPC proliferation and averting premature differentiation in C57BL/6 mice.

To test this idea, we purified primary MuSCs from both mouse strains for *in vitro* cell proliferation assays using EdU incorporation. Upon treatment with recombinant IGF1, new DNA synthesis in MuSCs/MPCs, isolated from both mouse strains, significantly increased by 2–3-fold (Fig. 4H). Consequently, we reasoned that the observed failure of muscle regeneration in BALB/c mice does not stem from an inherent deficiency in the proliferative ability of MuSCs/MPCs, for example in response to stimulation by IGF1. Rather, it appears to result from a deficiency in the secretion of pro-regenerative cytokines like IGF1 by macrophages, facilitating at least in part, the premature differentiation of MuSCs/MPCs and ultimately the failure of muscle regeneration in BALB/c mice following hindlimb ischemia. Indeed, recent research corroborates this perspective, illustrating that the administration of IGF1, via AAV9, significantly improved muscle mass and function post-HLI in BALB/c mice [64]. These findings highlight the potential of cytokines such as IGF1 to foster muscle regeneration in ischemic limbs, by promoting MuSCs/MPCs proliferation. Notably the beneficial pro-regenerative effects of cytokines such as IGF1 are conspicuously absent in BALB/c mice given the predominance of pro-inflammatory macrophages observed in this strain following HLI.

### Disruption of MuSCs fate switch is associated with aberrant macrophage-MuSC signaling crosstalk in human CLTI

Finally, we sought to translate our findings from murine models of HLI to human CLTI patients. We found that the distal muscle samples of three CLTI patients analyzed by scRNA-seq contained fewer MuSCs/MPCs compared to matched proximal samples (Additional file 1: Fig S5A). To further explore these findings, we conducted Pax7 immunostaining on cross-sections of limb muscles collected from an additional seven CLTI patients (Additional file 1: Fig S5B, Table 1). In four out of the seven patients, the number of Pax7+ MuSCs in the ischemic distal muscle were ~10–60% less than those in the matched non-ischemic proximal muscle (Additional file 1: Fig S5C). These results indicate that in at least a subset of CLTI patients (approximately 57–67%), ischemic-damaged limb muscle contains fewer MuSCs/MPCs compared to non-ischemic muscle.

**Fig. 5:**
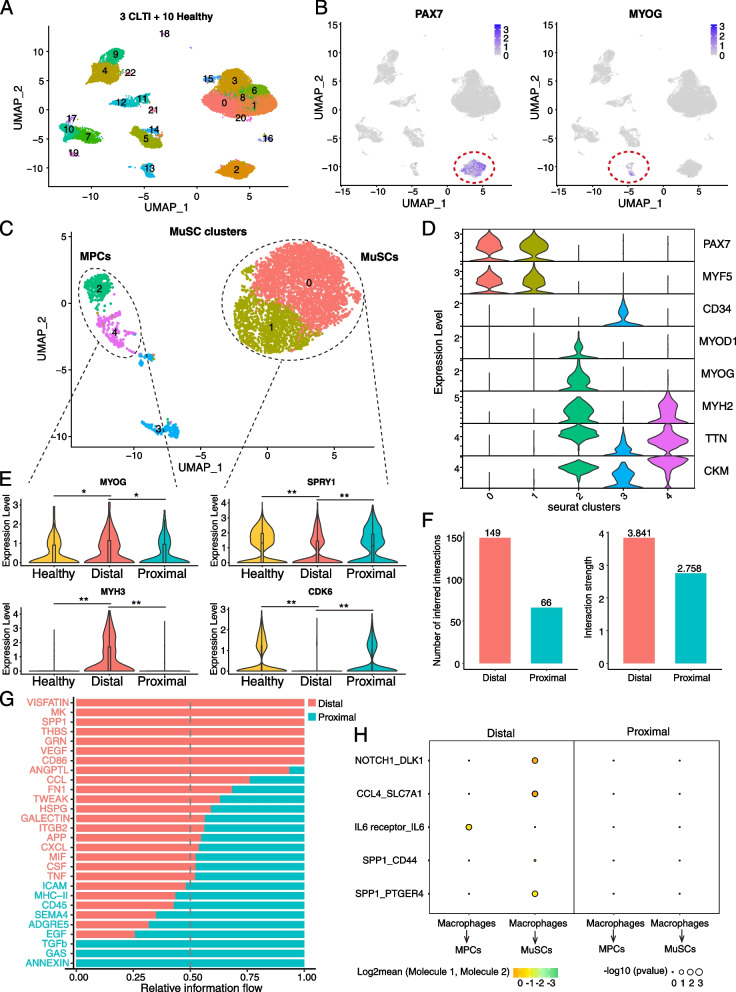
Increased pro-inflammatory signaling pathways between macrophages and MuSC/MPCs is associated with premature differentiation phenotype of MuSC/MPCs in the ischemia-damaged human limb of CLTI patients. **A**, **B** UMAP visualization presenting single-cell data gathered from human skeletal muscle tissue samples encompassing 3 CLTI patients under both proximal and distal conditions, along with data from 10 healthy individuals. **A** A total of 34,950 cells are represented in the UMAP graph. **B** The cells in clusters 2 and 13, illustrated in purple, exhibit Pax7 (left) and MyoG (right) expression respectively. **C** Cells from Pax7+ and MyoG+ clusters delineated in **B** are extracted from total cells and projected with an increased resolution onto a separate UMAP space that contain 5 sub-clusters (cluster 0–4), depicted in different colors. **D** Within this refined UMAP representation in **D**, cells in clusters 2 and 4 are denoted as “MPC”, demonstrating the expression of myogenic differentiation markers like MyoG and CKM. Conversely, the cells congregated in clusters 0 and 1 are identified as MuSCs, characterized by the expression of quiescent MuSCs markers Pax7 and Myf5. **E** The MPC and MuSCs defined in **D** are separated into healthy (yellow), distal (red), and proximal (blue) conditions for gene expression analyses. Violin plots show the expression of MyoG, MYH3, SPRY1, and CKD6 expression in MPCs (left) and in MuSCs (right), respectively. *P-value < 0.05; **P-value < 0.01. *P*-values were calculated by the Wilcoxon rank-sum test. **F** The numbers (left) and interaction strength (right) of inferred signaling interactions calculated by CellChat between macrophages and MuSCs in distal (pink, ischemic) and proximal (blue, non-ischemic) muscles of CLTI patients. **G** The significant signaling pathways between macrophages and MuSCs/MPCs are ranked based on their inferred strength differences between distal (ischemic) and proximal (non-ischemic) skeletal muscles. Signaling pathways colored in red are enriched in distal muscle, while those colored in blue are enriched in proximal tissues between macrophages and MuSCs/MPCs. **H** Ligand-receptor interactions inferred by CellPhoneDB between macrophages and MPCs or MuSCs in distal (ischemic) and proximal (non-ischemic) conditions. In distal conditions, we observed stronger pro-inflammatory signaling pathways, such as IL6, CCL4, and SPP1, compared to proximal conditions

Furthermore, to assess changes in gene expression and signaling activity of MuSCs/MPCs in human CLTI, we performed an integrative analysis of our human CLTI scRNA-seq data in conjunction with published human muscle scRNA-seq data generated from ten healthy individuals [65] (Fig. 5A, Additional file 1: Fig S5D). This analysis revealed Pax7+ MuSCs (cluster 2), MyoG+ MPCs (cluster 13) populations in human muscle, and a C3AR1+ macrophage population (cluster 7) (Fig. 5A, 5B; Additional file 1: Fig S5E). Incorporating data from healthy human skeletal muscles allows us to benchmark gene expression patterns of MuSCs/MPCs observed in CLTI patients against those in healthy individuals. To further elucidate the alterations in gene expression in MuSCs/MPCs in human CLTI, we segregated all the MuSC/MPCs from healthy and CLTI individuals onto a distinct UMAP space with increased resolution (Fig. 5C, Additional file 1: Fig S5F, S5G). Within this new UMPA space, we identified two major populations: Pax7+ MuSCs and MPCs committed to myogenic differentiation, which express *MyoG* or *CKM* (Fig. 5C, D). Importantly, the Pax7+ MuSCs from distal tissues exhibited substantially diminished levels of the quiescence/self-renewal marker *SPRY1*, as well as the cell cycle marker *CDK6*, when compared to their proximal counterparts and healthy MuSCs (Fig. 5E). This altered gene expression pattern hints at a decline in the self-renewal and proliferative capacity of MuSCs in the CLTI limbs affected by chronic ischemic damage. Additionally, the committed MPCs in distal tissues demonstrated significantly elevated levels of the early differentiation marker *MYOG* and terminal differentiation marker *MYH3*, compared to those in the proximal tissues of CLTI and in healthy muscles (Fig. 5E). These findings support the model that MuSCs/MPCs in the distal ischemic muscle of human CLTI patients are undergoing a loss of stem cell quiescence, a decline in cell proliferative capacity, and premature differentiation. These trends mirror the phenomena we observed in the murine CLTI model of HLI in BALB/c mice, thereby reinforcing the validity of our findings that premature differentiation of MuSCs/MPCs is associated with muscle regeneration failure in CLTI.

To further elucidate the underlying mechanisms associated with permanent tissue loss in CLTI patients, we investigated the potential association between the transcriptome signatures of MuSCs/MPCs and macrophages in our human CLTI scRNA-seq data. Leveraging intercellular communications inferred from scRNA-seq data [39], we found that the inter-cellular communication signaling pathways between macrophages and MuSCs/MPCs were markedly more pronounced in the ischemic muscle relative to the non-ischemic proximal muscle, both in terms of number and strength of signaling interactions (Fig. 5F). Notably, the top-ranked inter-cellular signaling flow from macrophages to MuSCs/MPCs in the distal tissue were characterized by well-documented pro-inflammatory pathways, included SPP1, CCL, TNF, and CXCL (Fig. 5G). Furthermore, we identified specific, significant pro-inflammatory signaling interactions, such as IL6-IL6R, CCL4-SLC7A1, and SPP1-CD44/PTGER4, exclusively in the distal tissue, while absent in the non-ischemic proximal tissues of CLTI between macrophages-MuSCs/MPCs (Fig. 5H). These observations suggest that the permanent tissue loss characteristic of human CLTI may be intricately associated with increased pro-inflammatory signaling interactions between macrophages and MuSC/MPCs within skeletal muscle damaged by chronic ischemia and that a persistent pro-inflammatory niche potentially disrupts skeletal muscle regeneration by altering the cell fate and functionality of MuSCs/MPCs.

## Discussion

In this study, we employed a well-controlled experimental approach, wherein muscle tissues were harvested from both the proximal (non-ischemic) and distal (ischemic) sections of amputated limbs from the same CLTI patients. This strategy notably diminishes the potential influence of pervasive confounding variables in human studies, such as diabetes, smoking, and age. Initial analysis of human CLTI scRNA-seq datasets unearthed a potential association between pro-inflammatory macrophages and the permanent tissue loss phenotype observed in CLTI. Further analysis revealed that muscle regeneration failure in CLTI might be closely tied to the premature differentiation and diminished proliferative capacity of MuSCs/MPCs in ischemia-afflicted limbs. Notably, these findings in human CLTI samples are supported by our results from murine models of HLI. Additionally, recent studies examining tissue samples from the gastrocnemius muscle in human PAD patients have shown a clear link between anti-inflammatory (CD206+) macrophages and an increase in both the number and size of MuSCs and muscle fibers [53]. While the observed connections between macrophage inflammation and the early differentiation of MuSCs/MPCs in both human CLTI and animal models are intriguing, we must recognize the potential constraints of our study. These include a limited sample size and the variable characteristics of individual patients. It is also important to note that the CLTI condition in humans is characterized by long-term ischemic effects, whereas the animal models mainly illustrate responses to short-term ischemia-induced tissue regeneration. Therefore, other cellular and molecular changes, including cell death, tissue fibrosis, and scarring, as well as unknown factors, may also play a significant role in the failure of muscle regeneration.

Furthermore, our findings suggest that the macrophages in the ischemic damaged tissue create an inflamed niche by secreted factors, which disrupts muscle regeneration by inducing precocious differentiation of MuSCs. Identifying the paracrine factors produced by the macrophages that mediate this effect may therefore throw light on potential therapeutic interventions. We have presented evidence that IGF1 signaling pathway, which is required for MuSCs/MPC proliferation, is lost in the ischemic damaged limbs of BALB/c mice. The critical role of IGF1 in promoting muscle regeneration upon ischemia injury is also supported by a recent study illustrating that the administration of IGF1 significantly improved muscle mass and function post-HLI in BALB/c mice [64]. Besides IGF1, our analysis identified several other pathways that demonstrate divergent activation patterns between macrophages and MuSCs in both the BALB/c and C57BL/6 strains. For example, we find that SPP1-CD44 signaling is highly active in macrophage-MuSC ligand-receptor pairs in both a murine CLTI model (BALB/c) and human CLTI patients. SPP1 is a pro-inflammatory cytokine that inhibits MuSC proliferation [66]. Hence, SPP1 signaling represents a potential mechanism to support our findings that the ischemic damaged MuSCs proceed to premature myogenic. Despite the identification of these candidate inter-cellular signaling pathways between macrophages and MuSCs/MPCs associated with muscle regeneration phenotype in CLTI, we would like to acknowledge that due to the complexity of the in vivo inflammatory responses and the dynamic and heterogeneous nature of both macrophage and MuSC/MPC populations [67], a thorough exploration of this aspect necessitates comprehensive functional and mechanistic studies to delineate the causal role of macrophages in regulating limb muscle regeneration in CLTI. We believe such functional and mechanistic investigation of these pathways in both engineered human muscle bundles [68] and the murine models of CLTI may shed light to develop therapeutic strategies to manipulate MuSCs and improve tissue repair.

## Conclusions

In summary, this study provides the single-cell transcriptome atlases of human CLTI and murine models. Our results represent a significant advancement in our collective understanding of the pathobiology of CLTI at the cellular, transcriptomic, and signaling levels. We show that the CLTI limb has a distinct pro-inflammatory macrophage signature and a MuSC phenotype hallmarked by premature differentiation. Our findings suggest new cellular mechanisms that can be potentially exploited to improve muscle function and lay a foundation for future muscle-specific therapies for limb salvage [69].

### Supplementary Information

**Additional file 1:** **Fig S1.** Single-cell transcriptome analysis of skeletal muscle in human CLTI patients. Related to Fig. 1. **Fig S2.** Single-cell RNA-seq atlas of limb muscle regeneration and damage in C57BL/6 and BALB/c mouse strains following HLI surgery. Related to Fig. 2.** Fig S3.** Distinct macrophage populations in C57BL/6 and BALB/c mice following limb ischemia. Related to Fig. 3.** Fig S4.** Single-cell analysis of MuSCs/MPCs in C57BL/6 and BALB/c mice before and after HLI surgery. Related to Fig. 4.** Fig S5.** Macrophage-MuSC cross talk in the ischemic limb of CLTI. Related to Fig. 5.

**Additional file 2:** **Table S1.** List of PCR primers used in this study.

**Additional file 3:** **Table S2.** The differentially expressed genes and their enriched GO terms in macrophage clusters 1 and 2 versus cluster 0 shown in Fig. 1E. Gene expression is quantified by scRNA-seq data. The GO table contains the gene listed used to calculate gene module score. Related to Fig. 1.

**Additional file 4:** **Table S3.** The top 20 marker genes for each sub-cluster of macrophages shown in Additional file 1: Figs S3A and B. Related to Fig. 3 and Additional file 1: Fig S3.

**Additional file 5:** **Table S4.** The differentially expressed genes and their enriched GO terms in macrophages purified from BALB/c versus C57BL6 mice at 3 days post HLI surgery. Gene expression is quantified by bulk RNA-seq. Related to Fig. 3.

## Data Availability

Published single-cell RNA-seq datasets from normal human skeletal muscle [65] were analyzed in our study. The datasets are available on the NCBI Gene Expression Omnibus (GEO) using accession number GSE143704: (https://www.ncbi.nlm.nih.gov/geo/query/acc.cgi?acc=GSE143704). Original sequencing datasets generated and analyzed in the present study, including murine and human CLTI single-cell RNA-seq data and macrophage bulk RNA-seq data, have been deposited in the NCBI Gene Expression Omnibus (GEO) and are available using the accession number GSE227077[69]: https://www.ncbi.nlm.nih.gov/geo/query/acc.cgi?acc=GSE227077.
