# Supplementary figures and images for: Skeletal muscle regeneration failure in ischemic-damaged limbs is associated with pro-inflammatory macrophages and premature differentiation of satellite cells
Source: Genome Med. 2023 Nov 10;15:95. doi: 10.1186/s13073-023-01250-y (PMC10636829; doi:10.1186/s13073-023-01250-y)

A

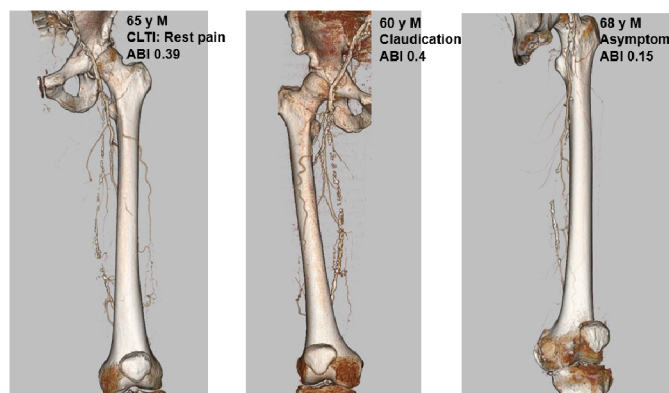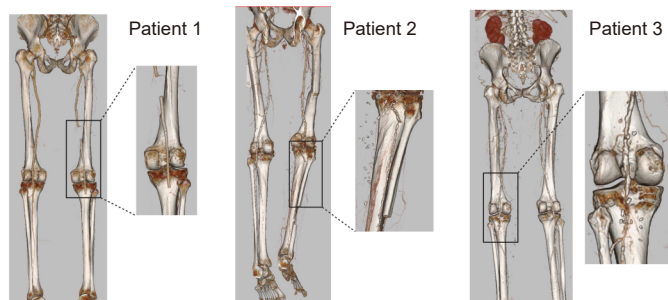

B

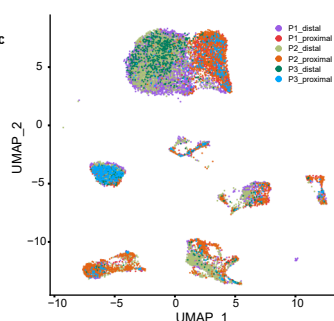

C

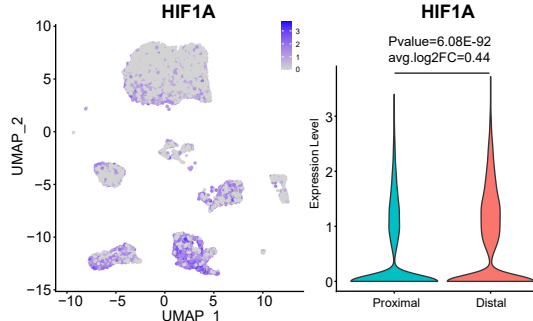

D

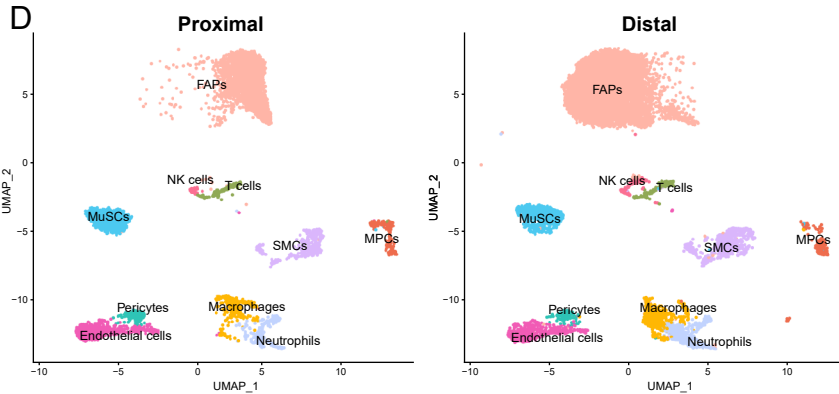

E

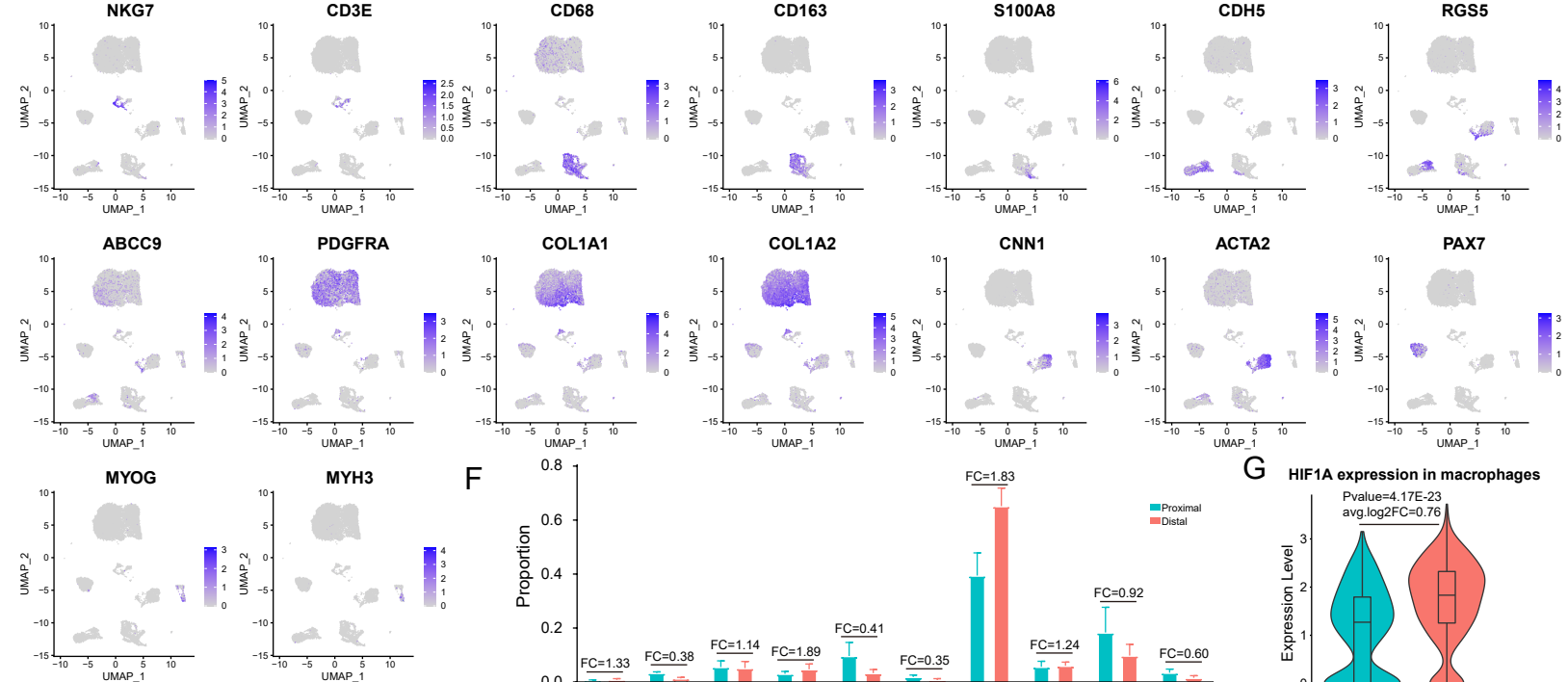

F

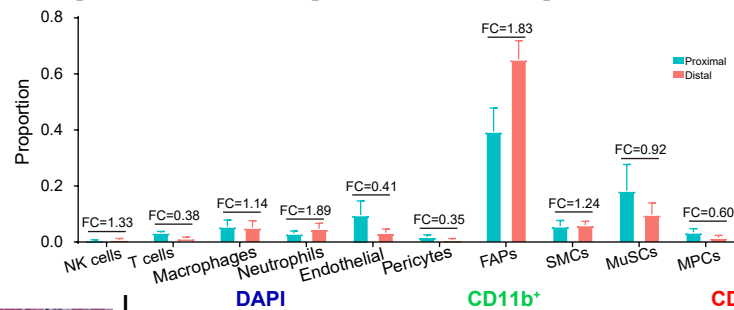

G

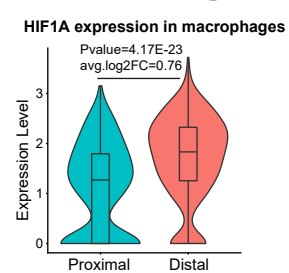

H

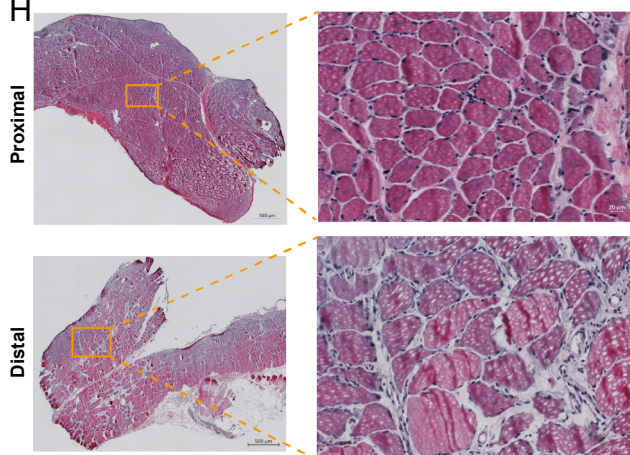

I

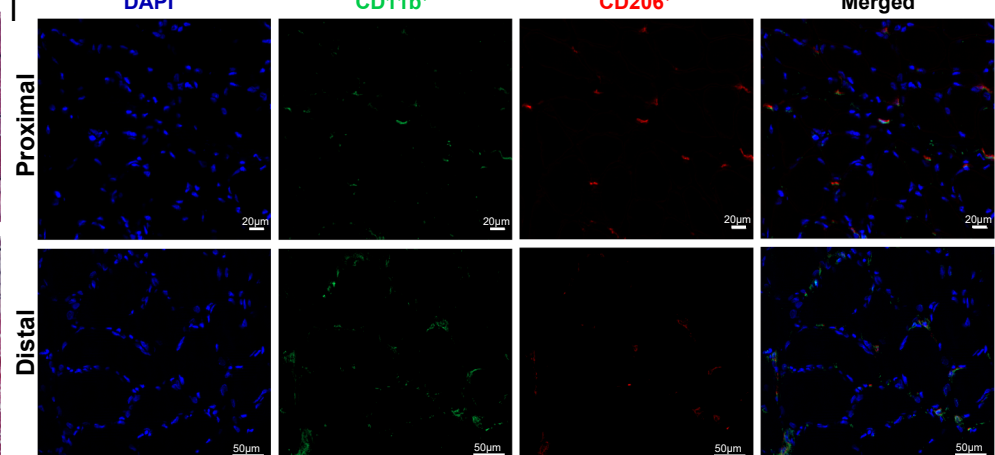

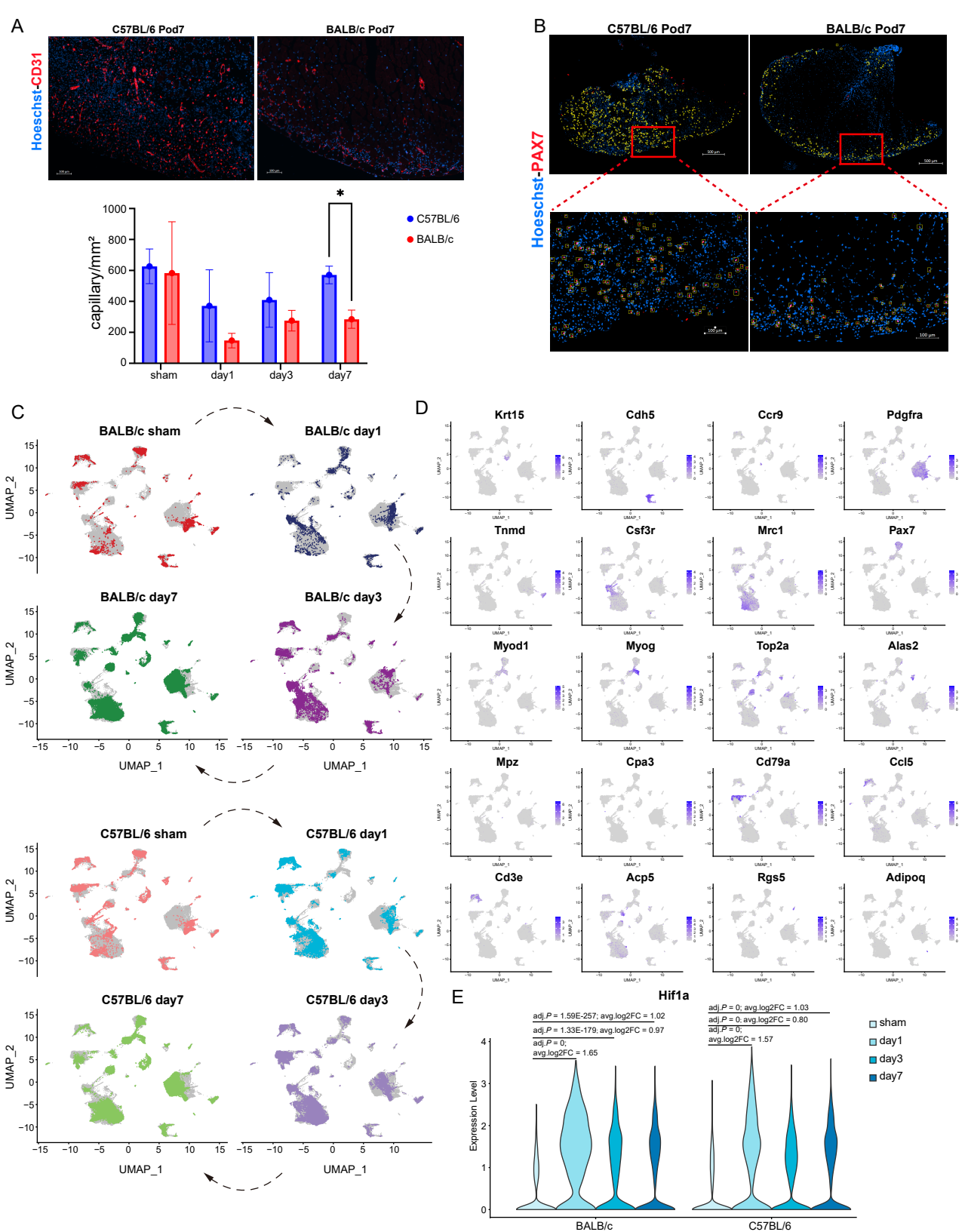

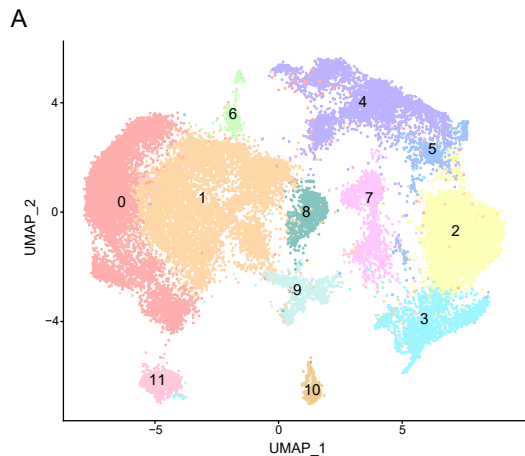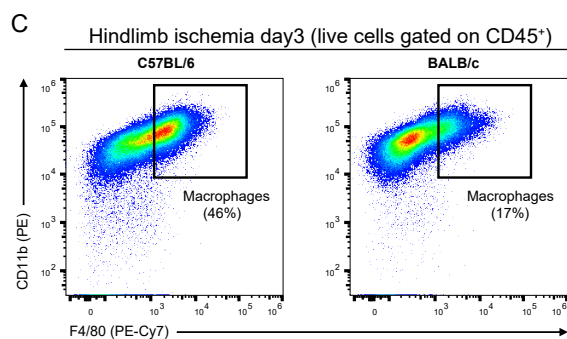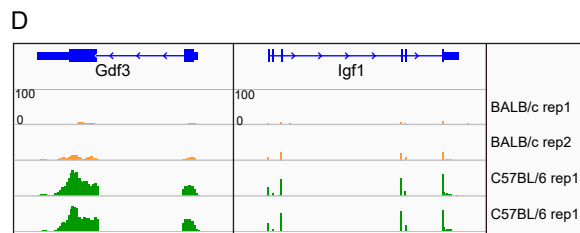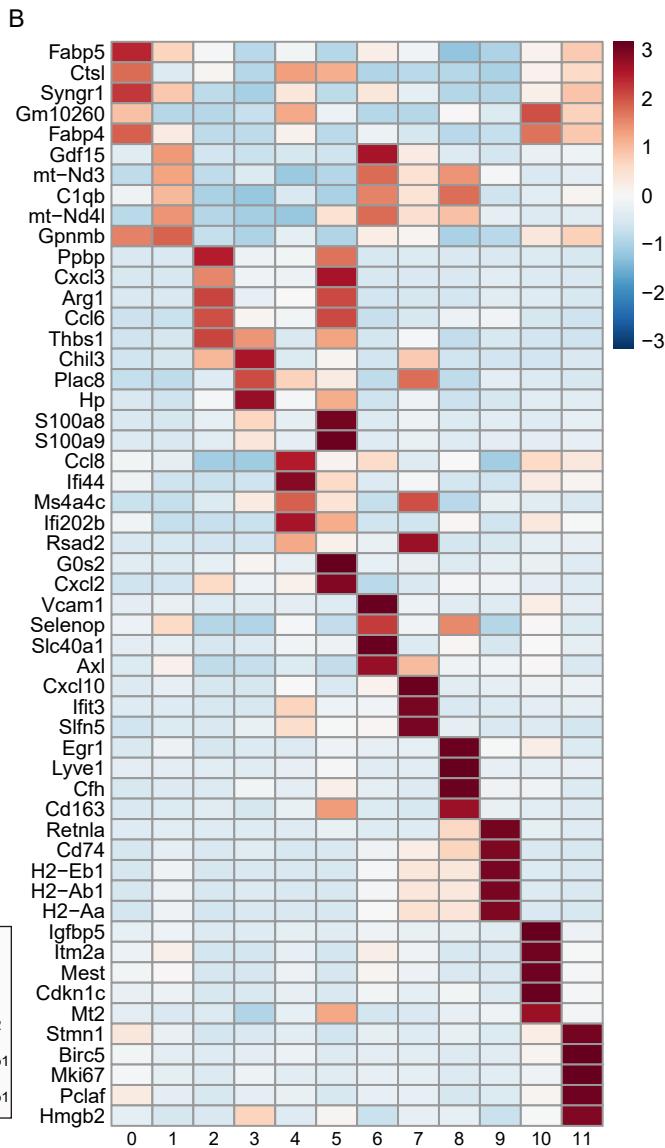

A

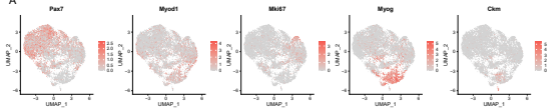

B

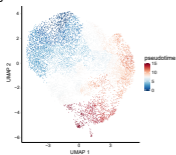

C

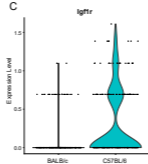

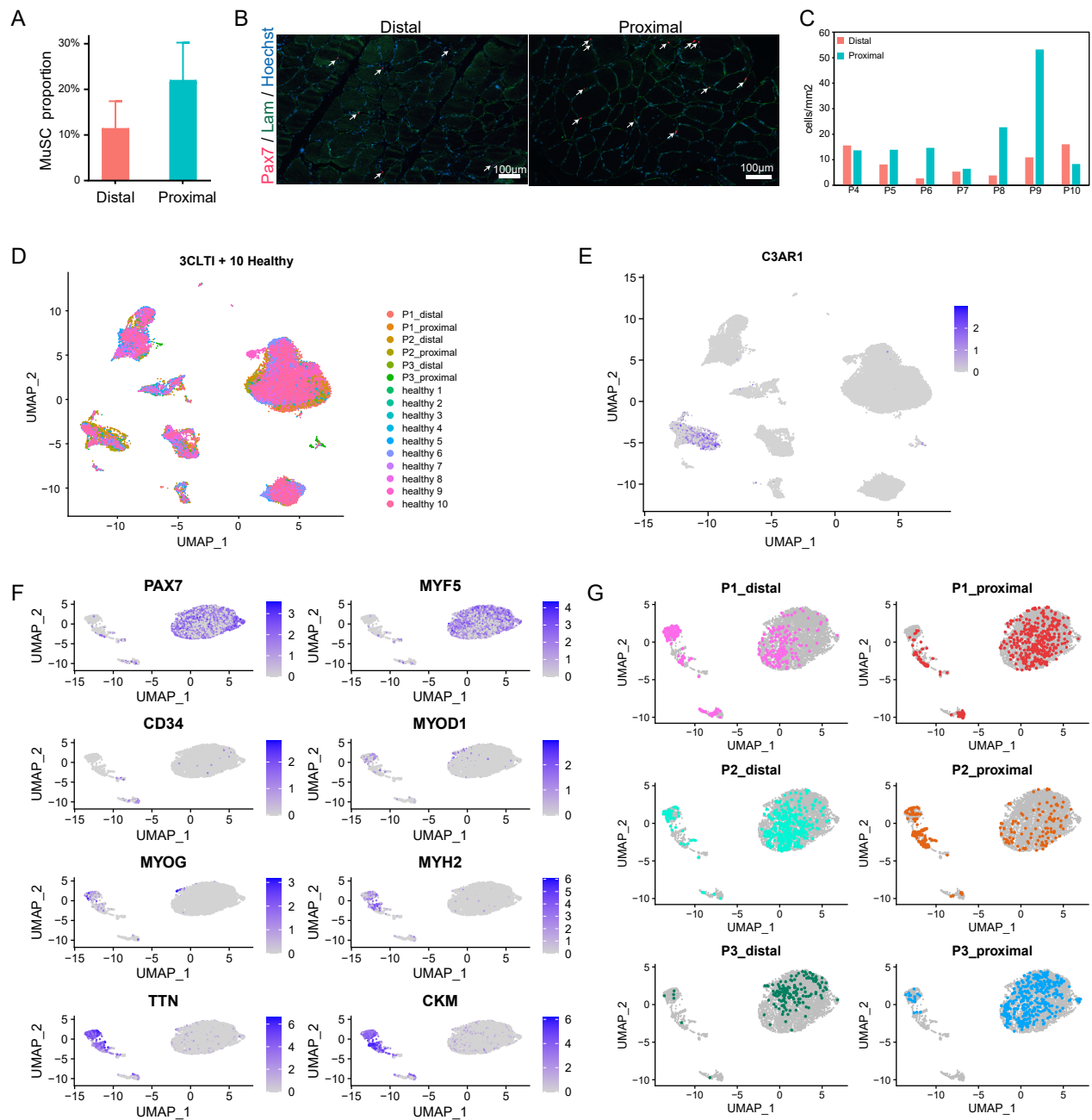

Supplement: Supplementary file 1 — Additional file 1: Fig S1. Single-cell transcriptome analysis of skeletal muscle in human CLTI patients. Related to Fig. 1. Fig S2. Single-cell RNA-seq atlas of limb muscle regeneration and damage in C57BL/6 and BALB/c mouse strains following HLI surgery. Related to Fig. 2. Fig S3. Distinct macrophage populations in C57BL/6 and BALB/c mice following limb ischemia. Related to Fig. 3. Fig S4. Single-cell analysis of MuSCs/MPCs in C57BL/6 and BALB/c mice before and after HLI surgery. Related to Fig. 4. Fig S5. Macrophage-MuSC cross talk in the ischemic limb of CLTI. Related to Fig. 5. [file 13073_2023_1250_MOESM1_ESM.pdf]
